# Current diagnostic and therapeutic management of chronic rhinosinusitis with nasal polyps in Austria: insights and unmet needs from a nationwide survey

**DOI:** 10.1038/s41598-025-07658-3

**Published:** 2025-07-02

**Authors:** Katharina Walla, Michael Habenbacher, Janina Kay, Laura Walrave, Philipp Günzl, Peter Valentin Tomazic, Alexandros Andrianakis

**Affiliations:** 1 Department of Otorhinolaryngology, Medical University of Graz, Graz, Austria; 2 GlaxoSmithKline, Vienna, Austria; 3 GlaxoSmithKline, Wavre, Belgium

**Keywords:** CRSwNP, Biologics, Endoscopic sinus surgery, Corticosteroids, Survey, Respiratory signs and symptoms, Health care, Respiratory tract diseases

## Abstract

This article aims to describe the current diagnostic and therapeutic practices for managing chronic rhinosinusitis with nasal polyps (CRSwNP) among Austrian ENT specialists. A cross-sectional, nationwide survey was conducted between November and December 2022 in Austria. A total of 50 ENT specialists, evenly split between hospital- and office-based physicians, participated. The questionnaire covered demographics, diagnostic and therapeutic approaches. CT imaging, nasal endoscopy, and blood eosinophil count were the most utilized diagnostic tools. Most participants applied the SNOT-22 for patient-reported outcomes. Local corticosteroids were the most frequently prescribed treatment. Systemic corticosteroid overuse and limited biologic adoption were noted. Hospital-based physicians managed significantly more patients with biologics. The initiation of biologic therapy was considered most appropriate following one FESS by the vast majority of respondents. Adherence to guideline-based evaluation criteria for biologic treatment response was suboptimal, highlighting gaps in clinical practice. Our survey highlights strengths in guideline adherence and areas for improvement, particularly in the diagnostic approach, systemic corticosteroid usage, and biologic treatment. Addressing educational gaps and refining clinical practices could enhance patient outcomes, reduce invasive procedures, and optimize resource utilization in CRSwNP management.

## Introduction

Chronic rhinosinusitis with nasal polyps (CRSwNP) is a chronic inflammation of the sinonasal mucosa characterized by typical rhinologic symptoms including nasal blockage, rhinorrhea, facial pain and impaired sense of smell^1^. The prevalence in Western countries has been estimated at 2–4%^2,3^. CRSwNP commonly displays a type 2 inflammatory profile in western countries, marked by elevated levels of IL-4, IL-5, and IL-13, along with eosinophilic infiltration^4,5^. Asthma bronchiale and non-steroidal anti-inflammatory drug-exacerbated respiratory disease (N-ERD), often coexist as comorbidities due to shared pathomechanisms. The presence of these comorbidities is linked to a higher disease burden and a greater risk of CRSwNP recurrence^6,7^. Diagnosis of CRSwNP is typically based on both clinical presentation and objective findings, including nasal endoscopy and computed tomography (CT) imaging, which reveal the characteristic nasal polyp formations. Given the chronic nature of the condition and the frequent need for individualized treatment, management often requires a combination of medical and surgical approaches. Traditional treatments for CRSwNP include local corticosteroids (LCS), systemic corticosteroids (SCS), and functional endoscopic sinus surgery (FESS)^1^. While LCS as first-line treatment are used to manage inflammation locally^1,8^, SCS may be employed in short-term bursts to provide more immediate symptom relief, though with potential side effects^1,9^. FESS is indicated when appropriate medical therapies fail to control symptoms sufficiently. However, a substantial proportion of patients experience disease recurrence^10,11^. Recent therapeutic advancements introduced biologic treatments targeting key cytokines involved in the inflammatory process of CRSwNP. Currently approved biologics for the treatment of CRSwNP include dupilumab, mepolizumab, and omalizumab^12^. These agents have shown promising results for efficacy and safety in clinical trials^13^ as well as in real life studies^14–16^, leading to their incorporation in treatment guidelines with established criteria for indication and evaluation^1^. However, several factors still need consideration such as the high cost and the (potential) lifelong treatment duration. Therefore, it is important to select the right candidate for biological therapy, as a considerable proportion of patients do not respond well to the treatment^17^. Moreover, certain questions remain open, like which biologic is most suited for which patient, which biomarkers are suitable as predictors for treatment response or are there any long-term side effects^18^.

Recent nationwide surveys on CRSwNP management have underscored how management practices vary across countries, reflecting differences in healthcare infrastructure, accessibility to biologics, practice patterns and adherence to guidelines^19–23^. In Austria, there is limited research on the current state of CRSwNP management, making it essential to examine how ENT specialists approach diagnosis and treatment of CRSwNP. Understanding these practices can provide valuable insights into areas where healthcare support systems, educational campaigns or national guidelines may need to evolve, particularly concerning the recently introduced biologic treatment. Therefore, this article aimed to describe the current state of CRSwNP management among ENT specialists in Austria, with a focus on diagnostic and therapeutic approaches.

## Materials and methods

### Study design and participants

A cross-sectional, nationwide survey was conducted to assess current diagnostic and therapeutic practices for CRSwNP among Austrian ENT specialists. The questionnaire was administered through online interviews or telephone calls between November 14 and December 2, 2022. A total of 50 ENT specialists, evenly split between hospitals (*n* = 25) and private practices (*n* = 25) across Austria, participated in this survey. The inclusion criteria required participants to be active ENT physicians treating CRSwNP patients. Selection followed a purposive sampling strategy to ensure a balanced representation of practice types and regions. Participation was voluntary and continued until the target sample size was reached.

### Study questionnaire

The survey was divided into four sections: (1) Demographics and practice characteristics: years of experience, type of practice (hospital or private office), number of treated CRSwNP patients in the last 6 months, and working location; (2) Diagnostic management of CRSwNP: Participants were asked about the usual diagnostic pathway, which diagnostic tools they use, and which patient-reported outcome measures (PROMs) they apply. (3) Treatment practices for CRSwNP: this section included questions on the most common treatments for CRSwNP, including LCS, SCS, FESS, and biologic therapy; (4) Comorbidities and interdisciplinary care: prevalence of relevant comorbidities and interdisciplinary collaborations were assessed. Question types varied between single-choice, multiple-choice, proportional-scales, and open-text responses. The questionnaire was developed by the author team based on current clinical guidelines and expert knowledge, followed by a pilot test involving six ENT specialists to ensure content validity. Based on their feedback, minor modifications were made. Due to the descriptive and exploratory nature of the survey, and because most items were structured and factual in nature, formal psychometric reliability testing was not conducted.

### Data analysis

SPSS © statistical software, version 29.0 (IBM ©, Armonk, NY, USA) was used. Quantitative data were reported using descriptive statistics. For the exploratory subgroup analyses (hospital-based vs. office-based), Whitney-U-Test was utilized to compare results of proportional-scaled questions (Rosenthal’s r was applied as effect size). χ^2^-test was used to compare categorical data (effect size was expressed with Cramer’s V). In contingency tables greater than 2 × 2, Bonferroni-adjusted Z-tests were used as post hoc test in case of significant χ²-test. Statistical significance level α was set at *p* < 0.05, two sided.

### Sample size calculation and power analysis

According to publicly available records, the total population of ENT specialists in Austria in 2022 was 795. Based on a desired confidence level of 80% and a margin of error of 10%, a minimum sample size of 39 participants would be sufficient for generalizable survey estimates (Sample size calculator, SurveyMonkey©). Our final sample consisted of 50 participants, exceeding this threshold and thus ensuring a robust representation of the national ENT specialist population. Given the exploratory nature of our subgroup analyses, we conducted post-hoc power analyses, given α-level, sample size and effect size (G*Power, Version 3.1.).

### Ethics

The survey was carried out in accordance with relevant guidelines and regulations. Ethical approval for the survey was waived by the Institutional Review Board of the Medical University of Graz, as the data were fully anonymized. Informed consent was obtained from all participants.

## Results

### Survey participants

A total of 50 ENT specialists from Austria participated in the survey, evenly divided between hospital-based (*n* = 25, 50%) and office-based settings (*n* = 25, 50%). The participants represented a broad range of professional experience, spanning from less than 5 years to over 30 years. The majority (62%, *n* = 31) of the ENT physicians were based in urban areas with a population exceeding 50,000 inhabitants. All participants had treated at least 10 patients with CRSwNP in the past 6 months, with 74% (*n* = 37) managing more than 30 patients during this period. Detailed data are given in Table 1.

**Table 1:** Demographics and characteristics of survey participants.

Characteristics	Total cohort *N* = 50	Hospital-based *n* = 25	Office-based *n* = 25	*p*-value
Years of experience				< 0.001*
0–5 years	11 (22%)	10 (40%) _a_	1 (4%) _b_	
6–10 years	15 (30%)	11 (44%) _a_	4 (16%) _b_	
11–20 years	8 (16%)	3 (12%) _a_	5 (20%) _a_	
21–30 years	11 (22%)	1 (4%) _a_	10 (40%) _b_	
> 30 years	5 (10%)	0 _a_	5 (20%) _b_	
Work location				0.690
< 10.000 inhabitants	4 (8%)	1 (4%)	3 (12%)	
10.000–50.000 inhabitants	15 (30%)	8 (32%)	7 (28%)	
> 50.000 inhabitants	31 (62%)	16 (64%)	15 (60%)	
Number of treated CRSwNP patients in the past 6 month				0.835
10–20	3 (6%)	2 (8%)	1 (4%)	
21–30	10 (20%)	5 (20%)	5 (20%)	
> 30	37 (74%)	18 (72%)	19 (76%)	

*Statistical significance at the Bonferroni-adjusted α-level. Each subscript letter (a, b) denotes a subset of practice-setting groups whose proportions do not differ significantly from each other at the adjusted significance level.

### Diagnostic pathway in CRSwNP

**Fig. 1:**
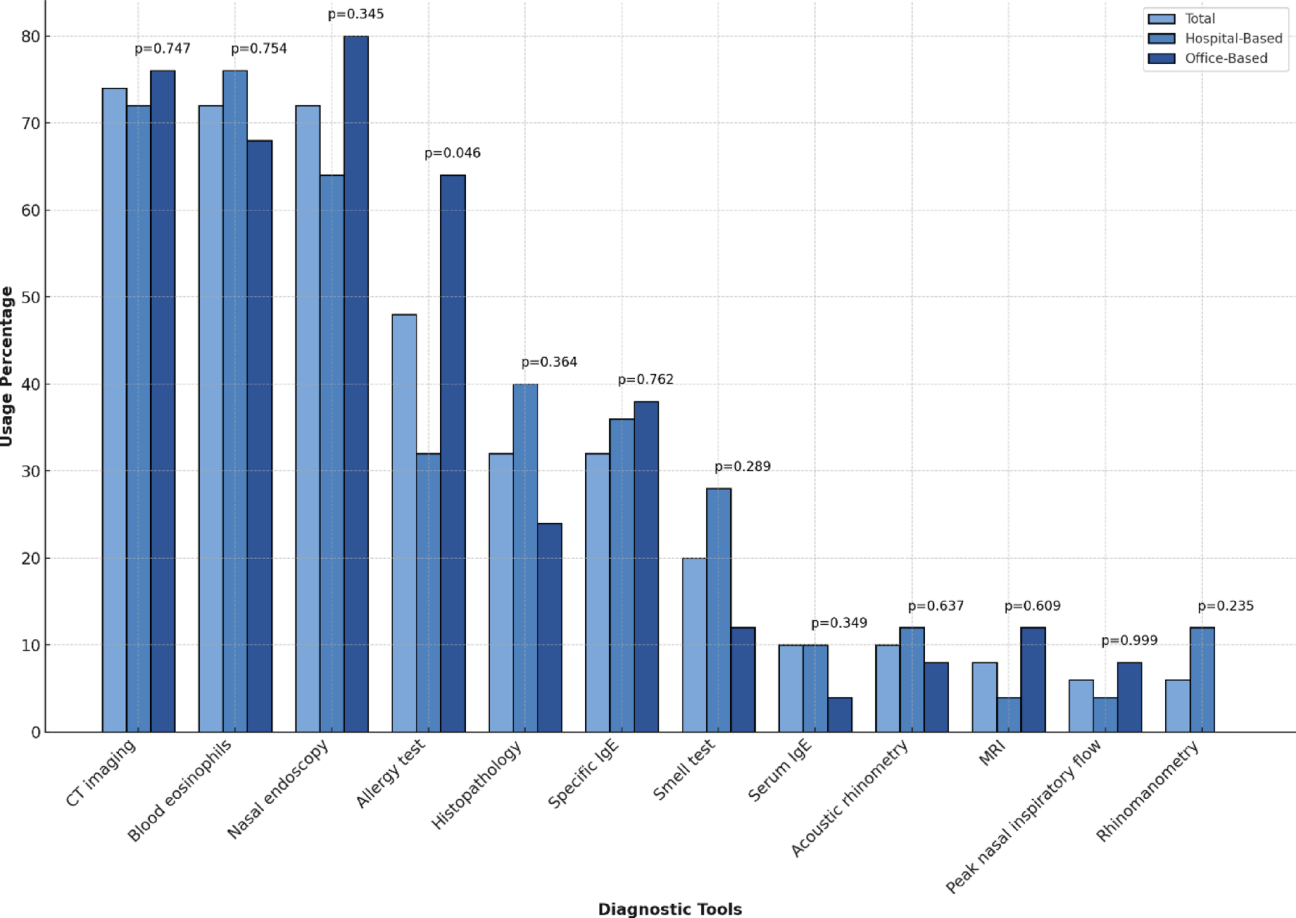
Response percentages for the multiple-choice question “Which diagnostic tools do you apply in CRSwNP management? History taking excluded.

ENT specialists reported that 96% of CRSwNP patients were referred by another physician, most frequently pulmonologists and primary care physicians, while the remaining 4% were seen without a referral. Half of the CRSwNP patients (50%) were pre-treated by primary care physicians before consulting an ENT specialist. By far the most frequently applied diagnostic tools were CT imaging (74%, *n* = 37), nasal endoscopy (72%, *n* = 36) and the assessment of blood eosinophil count (BEC) (72%, *n* = 36). Comprehensive results of diagnostic tool utilization are presented in Fig. 1. The most commonly used PROM was SNOT-22 (92%, *n* = 46), followed by overall symptom severity score (74%, *n* = 37), nasal congestion score (54%, *n* = 27), asthma-specific test like asthma control test or asthma quality of life questionnaire (34%, *n* = 17) and impaired sense of smell severity score (24%, *n* = 12).

### Treatment of CRSwNP

According to participants’ responses, 93% of CRSwNP patients received LCS, while 34% received SCS, 51% had FESS treatment and 11% were treated with biologics. Among the 51% with FESS treatment, 23% underwent more than one procedure. All participants (100%, *n* = 50) regarded more than one revision FESS within 10 years as excessive. The majority of ENT physicians (84%, *n* = 42) prescribe SCS as a treatment option for CRSwNP, with 67% indicating a treatment duration of less than two weeks, 31% reporting a duration of 2–4 weeks, and 3% extending treatment to 4–8 weeks. A dosage of 2.5–7.5 mg prednisolone-equivalent per day was reported in 60% of cases. On average, 49% of CRSwNP patients received < 1 SCS course per year, while 28% received 1–2 courses and 22% received 3–4 courses annually. All participants (100%, *n* = 42) agreed that SCS prescriptions should be limited to the shortest duration needed. Adverse effects related to SCS therapy were reported by only 4% of participating physicians (high blood pressure and worsening of diabetes mellitus). Detailed data on SCS treatment patterns can be found in Table 2.

**Table 2:** Practice patterns of SCS treatment (*N* = 42).

	Total cohort *N* = 42	Hospital-based *n* = 21	Office-based *n* = 21	*p*-value
Duration of SCS treatment				0.006*
< 2 weeks	28 (67%)	10 (47%) _a_	18 (85%) _b_	
2–4 weeks	13 (31%)	11 (52%) _a_	2 (10%) _b_	
4–8 weeks	1 (2%)	0 (0%) _a_	1 (5%) _a_	
> 8 weeks	0	0	0	
Daily prednisolon-equivalent SCS dosage				0.105
< 2.5 mg	7 (16%)	2 (10%)	5 (24%)	
2.5 mg – 7.5 mg	25 (60%	16 (76%)	9 (43%)	
> 7.5 mg	10 (24%)	3 (14%)	7 (33%)	
Proportion of CRSwNP patients receiving *n* SCS courses on average per year				
< 1	49%	50%	48%	0.520
1–2	28%	29%	27%	0.548
3–4	22%	21%	24%	0.119
> 4	1%	1%	1%	1.000

*Statistical significance at the Bonferroni-adjusted α-level. Each subscript letter (a, b) denotes a subset of practice-setting groups whose proportions do not differ significantly from each other at the adjusted significance level.

Participants were asked to state known biologics approved for CRSwNP treatment as open-text response. All participants (100%) stated at least one biologic agent, while 3% (*n* = 6) stated more than one. The best-known biologic medication was Dupixent® (Dupilumab), named by 60% (*n* = 30), followed by Nucala® (Mepolizumab, 28%, *n* = 14) and Xolair® (Omalizumab 18%). Chosen reasons to prescribe biologics were: reduction of need for FESS (96%, *n* = 48), improvement of sense of smell (60%, *n* = 30), high surgical or anesthesiologic risk (56%, *n* = 28), reduction of SCS therapy (56%, *n* = 28) insufficient symptom control (20%, *n* = 10) and accordance with guidelines (12%, *n* = 6). The start of biologic therapy was considered most appropriate following one FESS by 98% (*n* = 49) of participants, prior to any FESS by 2% (*n* = 1) and by nobody (0%) following 2 or > 2 FESS. The most commonly preferred dosing interval of biologics was every 4 weeks (74%, *n* = 37), followed by 2-weeks (24%), while 2% (*n* = 1) let their patients decide for themselves. Response to biologics was most commonly assessed by improvement of sense of smell, (70%, *n* = 35), reduction of nasal polyp score (NPS) (58%, *n* = 29), reduction of nasal congestion score (30%, *n* = 15) and improvement of quality-of-life score (SNOT-22) (28%, *n* = 14). Comprehensive results on biologic treatment are depicted in Table 3.

**Table 3:** Survey results on biologic treatment of CRSwNP.

	Total cohort *N* = 50	Hospital-based *n* = 25	Private practice *n* = 25	*p*-value
Known biologics				
Dupilumab	30 (60%)	17 (68%)	13 (52%)	0.387
Mepolizumab	14 (28%)	6 (24%)	8 (32%)	0.757
Omalizumab	9 (18%)	3 (12%)	6 (24%)	0.463
Reasons to initiate biologic treatment				
Reduction of the need for FESS	48 (96%)	25 (100%)	23 (92%)	0.490
Improvement of smell function	30 (60%)	15 (60%)	15 (60%)	1.000
Reduction of SCS usage	28 (56%)	12 (48%)	16 (64%)	0.393
High risk for surgery	28 (56%)	11 (44%)	17 (68%)	0.154
Insufficient symptom control	10 (20%)	8 (32%)	2 (8%)	0.074
Accordance to guidelines	6 (12%)	4 (16%)	2 (8%)	0.667
Preferred dosing interval				0.598
2 weeks	12 (24%)	6 (24%)	6 (24%)	
4 weeks	37 (74%)	19 (76%	18 (72%)	
Patients’ preference	1 (2%)	0%	1 (2%)	
Assessed criteria for biologic effectiveness				
Improved sense of smell	35 (70%)	14 (56%)	21 (84%)	0.062
Improved quality of life (SNOT-22)	14 (28%)	14 (28%)	14 (28%)	1.000
Reduction of nasal polyp score	29 (58%)	16 (64%)	13 (52%)	0.567
Reduction of nasal congestion score	15 (30%)	10 (40%)	5 (20%)	0.217
No need of SCS	1 (2%)	1 (4%)	0 (%)	0.312
No need of FESS	2 (4%)	1 (4%)	1 (4%)	1.000
Improved comorbidities	1 (2%)	0	1 (2%)	0.658

### Comparison between hospital-based and office-based settings

While many practices were consistent across hospital and office-based ENT specialists, few notable differences emerged: Referrals by pulmonologists were more prevalent in hospital settings (80%) compared to office-based settings (68%), while referrals by general practitioners were more frequent in office-based settings (96%) compared to hospital settings (4%) Hospital-based physicians managed significantly more CRSwNP patients who were treated with biologics, compared to office-based specialists (13% vs. 8%, *p* = 0.021, *r* = 0.33, power = 0.80). Moreover, they were significantly more likely to prescribe SCS for longer durations (*p* = 0.006, V = 0.47, power = 0.86). Detailed results on setting comparisons are presented in Tables 1, 2 and 3.

### Comorbidities and interdisciplinary aspects

The most commonly reported comorbidity in CRSwNP was allergic rhinitis (35%), followed by asthma (28%) and N-ERD (16%). Collaboration with pulmonologists was the most frequent form of interdisciplinary care with 86% (*n* = 43), followed by allergologists (8%, *n* = 16) and dermatologists (12%, *n* = 6).

## Discussion

Our survey provides key insights on the diagnostic and therapeutic practices for the management of CRSwNP patients among Austrian otorhinolaryngologists, shedding light on adherence to guidelines, the utilization of diagnostic tools, treatment approaches and the incorporation of advanced biologic therapies.

Austria’s healthcare system allows patients to access contracted health care providers easily at all levels. General practitioners are often the first point of contact; however, they don’t act formally as gatekeepers, so patients can see most specialists, including ENT, directly without a referral^24^. Hospital-based specialists reported a higher proportion of referrals by pulmonologists compared to office-based specialists whereas referrals from general practitioners were far more common in office-based settings than in hospitals. This pattern likely reflects the structured referral pathways within Austria’s healthcare system, where hospitals often handle more complex cases initiated by pulmonologists, and office-based care typically begins with referrals from general practitioners. Additionally, half of the CRSwNP patients were pre-treated by primary care physicians before consulting an ENT specialist, indicating that initial management often starts outside specialized care. These findings underscore the importance of interdisciplinary collaboration and the need for clearer referral and increased awareness among primary care physicians to ensure timely and proper management of CRSwNP, thereby preventing the progression of the disease.

EPOS defines CRSwNP clinically by the presence of typical sinonasal symptoms alongside objective evidence of mucosal inflammation. As CRSwNP symptoms very often overlap with those of other rhinologic diseases like (non)allergic rhinitis, it can be challenging to distinguish between these conditions based on symptoms alone^1^. Adding endoscopy/imaging significantly improves diagnostic accuracy and specificity^25^. EPOS advises to use nasal endoscopy as a standard part of the diagnostic process and recommends CT imaging if symptoms continue and endoscopy remains abnormal after proper medical treatment^1^. In our survey, the key diagnostic tools CT imaging and nasal endoscopy were indeed mentioned most frequently. However, given their importance as standard diagnostic methods, the observed rates should ideally be even higher. In comparison, a survey from Spain reported that 98% of surveyed ENT specialists routinely use nasal endoscopy, and 74% rely on CT scans to support diagnosis^21^. An international survey from the Young Otolaryngologists of the International Federation of Oto-rhino-laryngological Societies (Yo-IFOS) revealed that 62% of the participants order a CT scan immediately upon endoscopic CRSwNP diagnosis^19^.

The next most commonly used diagnostic tool in our survey was the assessment of BEC, a biomarker for type-2 diseases. Another frequently-cited type-2 marker, total IgE, was marked far less by the participants. More and more ENT specialists recognize the recently introduced endotype-based classification system, which categorizes CRSwNP into distinct endotypes (type 2 and non-type 2) based on the molecular pathways driving mucosal inflammation. CRSwNP in the western world typically presents a type-2 inflammatory process within the nasal mucosa, which is characterized by the infiltration of eosinophils and locally elevated IgE^4,5^. Eosinophil count, either in blood serum or nasal mucosa tissue, and blood total IgE are increasingly used as biomarker for type 2 disease. A tissue eosinophil count of ≥ 10/hpf, BEC of ≥ 150 cells/mL and total IgE count of ≥ 100 kU/L currently serve as cut-off values for the “evidence of type-2” EPOS/EUFOREA criterion in the context of indicating biologic treatment in CRSwNP^18^. Recent research reported that BEC on its own can fulfill the evidence of type-2 criterion in 96.3%, while total IgE is required in only 2 − 5% to meet the criterion^26^. Moreover, high BEC may predict a good response to SCS treatment or biologicals and can thus play a valuable role in guiding therapy planning^27,28^. Furthermore, eosinophilic CRSwNP has been associated with a higher recurrence rate, hence, high BEC can be used to aid in patient counseling^29,30^. In addition, elevated BEC and total IgE levels are both strong predictors of coexisting asthma^6^. Increased total IgE levels can also help identify patients who may benefit from anti-IgE therapy^31^. Although the 2020 EPOS guidelines could not reach a clear consensus on recommending the routine measurement of blood EOS or total IgE in CRSwNP diagnosis^1^, the importance of these biomarkers has increased substantially over the past years. For patients receiving dupilumab, the most recent EPOS/EUFOREA update advises measuring BEC at one and three months after initiation of therapy, with more frequent assessments for those with high baseline BEC (> 500/mL). This recommendation arises due to the potential for dupilumab to cause transient hypereosinophilia within the first months, necessitating close monitoring to avoid potential organ damage^18^.

Less than half of the survey participants evaluated for allergic disease in CRSwNP with skin prick test or specific IgE assessment^32^. In comparison, the Yo-IFOS survey reported that 66% of respondents evaluate for potential allergic triggers—via skin or blood tests—when managing CRSwNP^19^. Although the relationship between allergy and CRSwNP is not fully elucidated, these diagnostic tools can be utilized to determine whether an allergic component may be contributing to CRSwNP^33^. The prevalence of inhalant allergy in CRSwNP was 31% in a large epidemiologic UK study^34^. Given that inhalant allergies are treatable traits, performing a skin prick test as part of routine clinical practice is advisable when the patient’s history suggests the presence of an allergic condition^35^. Smell testing is performed solely by a fifth of the participated ENT physicians in CRSwNP diagnosis. Olfactory dysfunction in CRSwNP is very common, affecting between 60% − 80% of patients^36,37^. However, many patients are either unaware of their olfactory impairment or unable to accurately evaluate its severity, making subjective testing insufficient^38^. To address this, smell tests like the Sniffin’ Sticks are additionally advised to objectively evaluate the extent of olfactory impairment^39^. Acoustic rhinometry, peak nasal inspiratory flow and rhinomanometry were mentioned the least in the survey. While these nasal patency tests objectively measure the nasal air flow, they offer little additional value for CRSwNP diagnosis^1^.

Several PROMs specifically designed for CRS management have been created, with the SNOT-22 currently being the most commonly utilized and recommended PROM for quality-of-life (QoL) assessment. Moreover, the SNOT-22 is also the recommended QoL instrument for the indication and evaluation in biological treatment of CRSwNP. Nearly all participants reported using routinely the SNOT-22 as a key tool in CRSwNP management, reflecting a good adherence to international guidelines (1). About half of ENT specialists in Spain reported routine use of the SNOT-22^21^. In contrast, only one-third of the participating ENT physicians reported using asthma-specific PROMs in the management of CRSwNP, a figure that may appear low given the high prevalence of comorbid asthma and its role as a key EPOS/EUFOREA response criterion for biological treatments (18). However, this limited use could be explained by the strong interdisciplinary collaboration observed. These findings align with trends seen in Spain and Germany, where interdisciplinary management is more commonly used to assess these domains^21,23^. The close partnership may reduce the reliance on PROMs within ENT practices, as pulmonologists likely take a leading role in assessing and monitoring asthma-related outcomes.

The survey findings also provide detailed insights into the therapeutic strategies used by Austrian ENT specialists for managing CRSwNP. LCS remain the cornerstone of treatment. The observed high rate in our survey aligns with their established role as a first-line, guideline-recommended intervention^1^. Similar results were reported from a German survey^23^. However, while LCS are effective for mild-to-moderate cases, more severe or refractory cases often require additional therapies. Our survey revealed that SCS were prescribed for about every third CRSwNP patient. A quite similar tendency was observed in Germany, where 52% of ENT physicians administer SCS^23^. A much higher SCS use of 80% was reported in a Spain survey^21^. SCS are known for their rapid anti-inflammatory effects, reducing polyp size and relieving nasal congestion. Despite these benefits, the transient nature of their effects and the associated risks of systemic side effects necessitate their careful and limited use regarding frequency and duration. According to 2020 EPOS guidelines, no more than two courses of SCS should be given per year to avoid cumulative side effects^1,40^. However, in clinical practice, SCS overuse is common^41^. The observed rate of SCS use in our survey indicates the need of awareness-efforts. The 2020 EPOS guidelines also recommend restricting SCS use to short-term courses^1,40^. In this context, the survey results show that most ENT specialists adhere to this recommendation. Hospital-based physicians were significantly more likely to prescribe longer SCS courses. This may reflect the complexity and severity of cases typically seen in hospital settings, where patients often present with more advanced or refractory disease. FESS represents an important therapeutic option for CRSwNP patients when medical treatment fails to provide adequate relief. The main aims of FESS in CRSwNP are the removal of diseased tissue, including nasal polyps, and to functionally open the sinus drainage pathways. According to EPOS, primary ESS is generally indicated for severe CRSwNP cases with symptoms that persist or recur despite comprehensive medical treatment, including LCS and usually one or more courses of SCS within the preceding two years^1,40^. The participating ENT physicians in Austria reported that approx. half of patients underwent FESS treatment. Similarly, in Spain, 49% of ENT specialists identified FESS as the primary option for managing uncontrolled CRSwNP^21^. Our observed survey findings of revision surgery rates match with a recent meta-analysis calculating an overall FESS revision rate of 18.6% in CRSwNP patients^42^. Repeated surgeries often increase the risk of complications, reduce the likelihood of success, and result in permanent scarring^10,11^. In this context, all survey participants agreed that more than one revision FESS within 10 years is excessive.

In the last years, newly introduced biologics have revolutionized the treatment landscape for recalcitrant CRSwNP, offering highly effective and personalized therapeutic options. Our survey revealed that hospital-based ENT specialists managed significantly more CRSwNP patients with biologic treatment than office-based physicians. Hospitals often serve as referral centers for more complex or severe cases and may also provide more experience with newer, cutting-edge therapies, such as biologics, due to the ongoing exposure to the latest clinical advancements. We have asked ENT physicians to state known biologics approved for CRSwNP treatment. Currently approved biologics in Austria are dupilumab, mepolizumab and omalizumab^12^. All participants stated at least one biologic agent, while only 3% were able to name more than one. It still remains elusive which biologic is best suited for which patient and reliable biomarkers are lacking. German ENT specialists reported that dupilumab was the most common prescribed biologic, followed by mepolizumab and omalizumab^23^. Similar results were observed in a national US survey^20^. Nevertheless, ENT physicians should be familiar with all approved medications. Some patients may not respond well to the prescribed biologic or experience medication side effects. In such cases, switching to an alternative biologic can be considered. Recent studies reported that 4−16% of patients required switching to an alternative biologic^43,44^. Austrian ENT physicians should therefore be more thoroughly informed about the various biologics and the option of switching biologic agents. In Austria, Dupilumab and Mepolizumab are approved as add-on therapies to LCS for adults with severe, recurrent CRSwNP who have undergone surgical treatment and either failed to respond, are intolerant, or have contraindications to SCS^45^. In contrast, Omalizumab is approved without requiring prior treatment with or contraindications to SCS^17^. The current EPOS/EUFOREA criteria suggest performing a complete FESS prior to initiating biologic therapy. However, it remains unclear what extent of surgery is necessary, whether revision surgery vs. biologics represents the superior approach or whether it is preferable to start biologic treatment with “clean sinuses” after surgery^18^. In our survey, the vast majority of ENT physicians considered initiating biologic therapy most appropriate after performing one FESS. Similar, in the German survey, 72% of the ENT specialists regarded one FESS as the required amount before initiating biologic therapy^23^. This high consensus underscores the perceived effectiveness of biologic treatments. Nevertheless, prospective studies are urgently required to address these unanswered questions with robust, evidence-based data. The approved dosing intervals for biologics vary, with Mepolizumab administered every 4 weeks, Dupilumab every 2 weeks, and Omalizumab offering flexibility between 2- and 4-week intervals depending on IgE levels^12^. In our survey, most ENT-physicians preferred the 4-weeks dosing interval. While it is understandable that a notable concern for patients is the discomfort associated with repeated injections, particularly as the therapy may need to be continued for life, a recent study demonstrated that tapering of dupilumab treatment up to a 6-week interval was possible in the majority of cases, when an initial good response is seen^46^. Tapering data for mepolizumab and omalizumab in CRSwNP treatment are currently not available. Further studies are urgently warranted to further examine the process of tapering in biologic treatment. The EPOS/EUFOREA expert panel provided current criteria for evaluation of response to biologics in 2023, which are reduced NPS of ≥ 1; SNOT-22 score of ≥ 40 or reduction of ≥ 12; improved sense of smell; no need of SCS/revision surgery; and reduced impact of comorbidities^18^. To qualify for reimbursement in Austria, the national Federation of Social Insurances requires solely a reduction in NPS of ≥ 2 for dupilumab, and ≥ 1 for mepolizumab and omalizumab within 6 months of treatment initiation^45^. The suboptimal use of response criteria in our survey might be attributed to several factors including insufficient awareness among physicians, gaps in professional trainings, limited consultation time, inconsistencies in local practice protocols, and reimbursement challenges.

The current survey has identified strengths in clinical practice as well as areas for improvement. To address these gaps, few targeted actions are warranted. Updated national guidelines and educational workshops might enhance the usage of key diagnostic tools, biomarkers and PROMs. A nationwide training program on biologic therapies may enhance ENT specialists’ familiarity with approved agents, eligibility criteria, and response assessment. Structured referral pathways and shared care models might improve interdisciplinary collaboration between ENT specialists, pulmonologists, and allergists, for a better disease management.

There are some limitations that need to be acknowledged. First, the survey relied on self-reported data, which may introduce recall bias and does not permit verification of responses. Second, although the sample size exceeded the calculated minimum for adequate representation, it may still limit the generalizability of the findings to all ENT specialists in Austria. Third, accidental or intentional bias in responses cannot be entirely ruled out. Despite these limitations, the study has several strengths. It is the first of its kind to provide a nationwide overview of CRSwNP management practices in Austria, offering a comprehensive analysis of both hospital- and office-based settings. Furthermore, the survey’s insights into the adoption of biologics offer a valuable foundation for future policy and educational initiatives aimed at optimizing patient outcomes. Given the dynamic nature of medical advancements, it would be beneficial to repeat this survey in a few years to track changes in practice patterns and evaluate the long-term impact of educational and policy interventions. Such a longitudinal approach could provide meaningful insights into emerging trends and further refine the strategies for CRSwNP management in Austria. Comparing future findings with the current baseline data will allow for a better understanding of progress and areas that still require attention.

## Conclusions

This report provides important insights into current practices for managing CRSwNP in Austria, identifying strengths in adherence to guidelines as well as areas for improvement. Key challenges include improving diagnostic approach, optimizing treatment strategies, increasing awareness of biologic therapies, and standardizing evaluation methods.

## Data Availability

The datasets generated during and analyzed during the current study are available from the corresponding author on reasonable request.
